# Towards a culturally competent health professional: a South African case study

**DOI:** 10.1186/s12909-018-1187-1

**Published:** 2018-05-22

**Authors:** Margaret Matthews, Jacqueline Van Wyk

**Affiliations:** School of Clinical Medicine, Nelson R Mandela School of Medicine, University of KwaZulu-Natal, Durban, Republic of South Africa

**Keywords:** Cultural competence, Medical education, Language, Culture, Intercultural communication

## Abstract

**Background:**

South Africa (SA) has a growing multilingual and multicultural population of approximately 55 million people, and faces service delivery challenges due to a shortage in skilled health professionals. Many health care facilities still depict distinct racial and ethnic characteristics that date back to the *apartheid* era, and there are reports of racial intolerance or preferential treatment at some facilities. There is limited literature in South Africa on cultural competence or on how to train health professionals to provide culturally competent care. This paper describes a study conducted to gain a better understanding of final year medical students’ perceptions regarding concepts related to cultural and linguistic competence in the SA healthcare setting.

**Methods:**

An exploratory, cross-sectional, analytical study used a questionnaire to collect data from final year students at the medical school.

**Results:**

The demographic profile indicated considerable diversity in the respondents for languages spoken, ethnicity and religion. Responses indicated a level of cultural awareness and, according to the Cross Framework, a position of cultural pre-competence. This position was supported by the majority expressing high levels of agreement with the items deemed to indicate responsiveness: a desire for cultural competence to be promoted in the medical curriculum and for professional development to improve delivery of services and support to linguistically and culturally diverse groups. No significant association was found when analysing the latter item against demographic grouping variables. However, although not significant, a diminishing trend emerged in the rankings of monolingualism, bilingualism and multilingualism, suggesting that the ability to speak more than one language could possibly be a facilitating factor in acquiring cultural competence.

**Conclusions:**

In response, it is recommended that specific learning objectives be included in the medical curriculum. Understanding of concepts related to both individual and institutional cultural competence would improve insights into their relevance in responding to the challenges related to culture in SA healthcare. Further research in teaching cultural competence is recommended. In order to respond to local needs, this should include research at a community level to analyse patients’ perspectives and satisfaction with the cultural competence of healthcare providers and organisations serving the SA public.

## Background

South Africa (SA) is home to a multilingual and multicultural population of approximately 55 million people. The diversity of the SA population is reflected in its language, religious beliefs and customs [1]. There are 11 official languages (and many unofficial ones) [2], and a range of religions, including Christianity, traditional African religions, Hinduism, Islam, Judaism and other smaller groups [3].

The past two decades has seen a rapid change in the socio-political landscape with increasing inequality and poverty in underserved communities. Concurrent with the rapid urbanisation of rural populations, an increase in migrants, mainly from other African countries, has resulted in approximately 35 million (63.6%) people now residing in urban areas [1]. At a social level, the country faces increased service delivery challenges due to the shortage in skilled health professionals (HPs) [4]. Many health care facilities, depending on their locations, still depict distinct racial and ethnic characteristics that date back to the *apartheid* era. Within this context, we find both anecdotal and reported evidence of racial intolerance, cases of preferential treatment, or language being used as an exclusionary measure at some healthcare facilities [5].

Internationally, a large body of literature has been devoted to cross-cultural challenges faced by healthcare professionals. Much has been published on ways of dealing with cultural differences and improving intercultural communication [6–12]. The increasing need for diversity training and cultural awareness extends beyond healthcare and includes all spheres of everyday life [13–15].

Culture is described as the *“integrated patterns of human behaviour that include the language, thoughts, communications, actions, customs, beliefs, values, and institutions of racial, ethnic, religious, or social groups”* [16, 17]. The concepts of cultural and linguistic competence in health are increasingly being viewed jointly [16], and refer to *“a set of congruent behaviours, attitudes, and policies that come together in a system, agency, or among professionals that enables effective work in cross-cultural situations”* [16].

Most South African studies have highlighted language as a major barrier to effective healthcare delivery [18–20]. Emanating from a uniquely *apartheid*-constructed society, in which HPs spoke mainly English whilst most patients spoke an African language, Crawford described a serious level of *“misapprehension, mistranslation, loss of meaning, and consequent misunderstanding that occurred on a daily basis between doctors, nurses and patients”* ([20] p.34). She also criticised the use of the Western biomedical model, and recommended a change towards *“a more culturally sensitive patient-centered model of care” (*[20], p.42). Levin described how “*language and cultural barriers were cited by more parents as a major barrier to health care than structural and socio-economic barriers”* ([18] p.1076). Culture-specific models of disease affect how illness is understood by many SA patients [21, 22]. In culture-bound syndromes, terms used to describe symptoms, or metaphors used for phenomena related to disease, do not always have an easy translation or equivalent English term [22]. Interpreters are not readily available in the public health sector, where many doctors, irrespective of their home languages, conduct medical interviews with the assistance of HP staff who act as untrained interpreters or cultural brokers when necessary [11].

Recommendations to address issues of non-concordance in language in SA have resulted in the implementation of basic communication skills training in local African languages at most medical schools [23] to facilitate some cross-cultural understanding and to foster respect for the culture of patients [24] . However, the effect and its magnitude are yet to be researched. Given the complexity of SA’s history and the need for effective healthcare, Burch questions whether institutions should focus efforts on cultural competence or language proficiency in training as a means to improve care [25].

Internationally, the notion of cultural competence has emerged as a strategy to address disparities in healthcare which may result from racial, ethnic and language differences. In an attempt to improve health outcomes, emphasis is placed on reducing cross-cultural misunderstandings through developing competencies in HPs to deal with issues related to culture in the consultation [9, 26–28].

Literature in the SA context on cultural competence is however limited, and there is little evidence on how best to train medical students to provide culturally competent care. Chipps et al. [29], in a systematic review of the literature on cultural competence training for HPs in community-based rehabilitation, questioned the relevance of international literature for use in the SA context. She argued that *“African people form the majority of the population but most health care professionals (including African health care professionals) have been trained in Western traditions of helping*” ([29] p93).

Cultural competence has been espoused as an organizational strategy to reduce disparities in healthcare [30] and various conceptual frameworks have been suggested for use in medical education [31]. Most research has been done in high income countries, focussing on the ability of how their systems and organisations provide healthcare to ethnic minorities. In those contexts, various tools have been developed to assess cultural competence of organisations and individuals. This is a complex and challenging task, and available tools have not always been validated for use in specific settings [32–34]. Self-assessment checklists are thus often used to promote discussion and to encourage self-reflection amongst individuals on their awareness and understanding of the influence of cultural norms, customs, religions, traditional versus alternative medicine, and other contextual cultural influences, including their own cultural backgrounds, on their interactions with their patients [34–36].

**Fig. 1:**
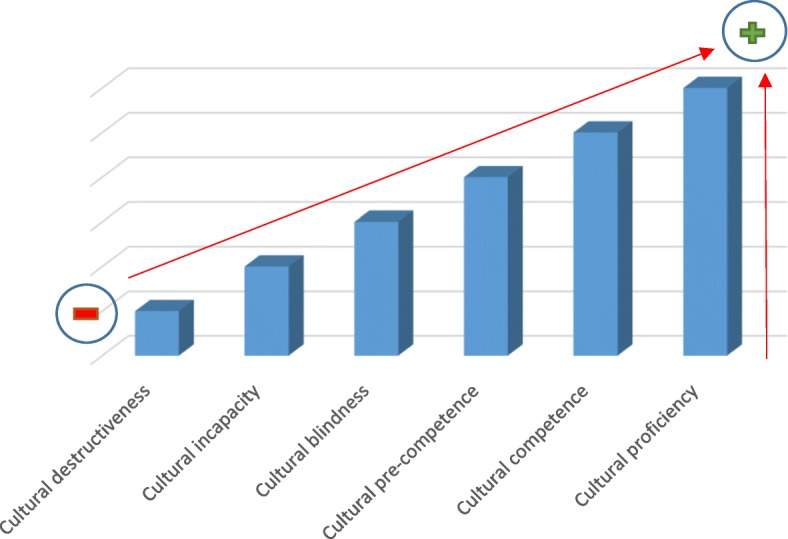
Cultural Competence Continuum (Cross, Bazron, Dennis & Isaacs 1989) [17] Source: Adapted by the authors from an image used by the National Center for Cultural Competence, Georgetown [34]

Cross et al. [17] describe cultural competence as a complex process which develops over a continuum in systems and organisations, with individuals at all levels of an agency, including policy makers, administrators, practitioners and consumers themselves, being expected to participate in the process. The Cross framework describes six main positions (see Fig. 1) in this continuum ranging from cultural destructiveness to cultural competence or proficiency. The most negative end of the continuum is represented by cultural destructiveness, which is a bigoted position in which a dominant culture disenfranchises, controls or exploits another group. The next position, referred to as cultural incapacity, implies a lack of capacity to help minority clients or communities, or the enforcement of racist policies. Cultural blindness reflects a well-intended liberal policy which treats all people in the same manner. Such services ignore cultural strengths and encourage assimilation. Cultural pre-competence implies a responsive position in which there is an appreciation of the existing weaknesses in treating specific populations. In such a position, it is considered important that professionals from minority groups receive training in *“the function of culture and its impact on client populations”(*[17] p.17). The most positive end of the continuum is advanced cultural competence or proficiency. The latter is characterised by culture being held in high esteem with advocacy for cultural competence throughout the system and for the improvement of relations between the various cultures within the society. It is imperative that development takes place at all levels and that five main elements are emphasized for a system, institution or agency to become proficient or reach an advanced stage of cultural competence. The elements are valuing diversity, the performance of cultural self-assessment, consciousness of the dynamics of cultural interaction, institutionalisation of cultural knowledge, and adapting to diversity [17].

As health professions educators, it is important to identify what is required of medical students to be culturally competent practitioners upon graduating from programmes. Training should be directed towards knowledge about culture (facts and cultural traits), specific skills (behaviours), awareness about self and others, and the development of cultural intelligence (including linguistic, spatial, intra- and inter-personal intelligence) [37] .It should also provide appropriate opportunities for engagements to bring new insights into their own mental processes, convictions and behaviours and enable transformative learning through a revision of their own beliefs and behaviour [38].

Despite efforts to sensitise medical students to the importance of cultural issues in SA healthcare, cultural competency training and assessment are not comprehensively addressed in most SA medical education programmes. At an individual level, cultural competence is not considered a critical competency for graduating doctors. At this and other SA medical schools, teaching and learning related to cultural issues in health has been subsumed in teaching activities in communication skills, longitudinal community attachments in Family Medicine or the medical humanities (academic staff at three SA medical schools, 20th – 23rd February, 2018, personal communications). In addition, healthcare facilities in the public health sector which provide the training platform generally do not incorporate principles of cultural competence, which are regarded as important for learning and for delivering quality health care [39].

Whilst a previous study with medical students at the same institution [40] highlighted the need to improve their ability to deal with cultural challenges, this study was conceived to explore the current status of their perceptions of concepts of cultural and linguistic competence in the SA healthcare setting. The study was thus conducted at an individual rather than an institutional level. The study aimed to gain an understanding of the phenomenon in context by obtaining a current snapshot view of medical students’ perceptions. The specific objectives are expanded upon in the methodology section below. The study is considered important to contribute to curriculum development and to raise awareness in the SA health context of the importance of individual cultural competence within a culturally competent health system, as part of professionalism and quality clinical care. The study aims to contribute to educators’ understanding of medical students’ needs and to contribute to transformation of the curriculum to respond to the socio-cultural needs of local societies [41]. It also hopes to address the divisions of the past and improve social cohesion in healthcare settings [40]. The ultimate aim is to provide training that produces practitioners that are able to bridge the gap between Western and other, mainly African, worldviews when treating patients.

## Methods

The study was an exploratory cross-sectional analytical study of the final year student cohort at the Nelson R. Mandela School of Medicine (NRMSM) of the University of KwaZulu-Natal (UKZN) in Durban (SA). A cross-sectional study design was used to eliminate selection bias.

The study population included all medical students in the final year in 2015. No sampling was applied and there were no exclusion criteria. This study population was the first cohort to be exposed to a revised communication skills course, offered in the first three years of the MBChB programme. The course emphasized a clinical method which focussed on gathering information from a three-part perspective; the biomedical, contextual and patient perspectives [42, 43]. Students were taught of the importance of patient- [39, 44] and person-centredness [45]. To improve intercultural communication, emphasis was placed on incorporating the patient’s ideas, concerns, cultural and religious beliefs, and expectations in the consultation [43], and on showing empathy and respect for the patient’s culture. The study population had also been exposed to patients in both urban and rural clinical settings, starting from their 3rd year of study. Other concepts related to culture were introduced at various academic levels and in some disciplines including Rural Health, Psychiatry and Paediatrics.

Using a self-administered questionnaire, the specific objectives explored:

1. The demographic details of the sample population: This was to analyse the diversity of the sample in relation to the specific objectives of the study. Categorical data was grouped using headings from the national SA Census results (2011).
2. Perceptions of cultural and linguistic competence: Various instruments used to heighten awareness of cultural competence in the healthcare context were reviewed for content. The search concentrated on instruments/ items which explored individual competence. The items were taken from validated self-assessment checklists used for promoting cultural and linguistic competency at the National Center for Cultural Competence at Georgetown University [36]. Items were reviewed and those items relevant to the SA context were included verbatim or adapted for readability. Specific items were added to explore students’ perceptions regarding language in SA healthcare. The questionnaire was checked for clarity of meaning by the researchers and piloted with fifth year students, and explanatory notes were added to aid students’ understanding e.g. to explain the term *“cultural broker”* (Refer Table 2).
3. Responsiveness of the students in terms of two specific items:
  - students’ expressed need for the curriculum to promote cultural competence, and
  - students’ expressed need for professional development to improve their ability in the provision of services to culturally and linguistically diverse groups.

Respondents were asked to indicate their degree of agreement with the individual items by using a 5 point Likert scale (A-Strongly Agree; B-Agree; C-Neutral; D- Disagree; E-Strongly Disagree). When using a Likert scale, central tendency, social desirability and acquiescence bias are possible forms of information bias. Central tendency bias can be limited by use of an even numbered Likert scale with no neutral, but generally, as in this case, it is preferred to include neutral as a legitimate opinion, which indicates mixed satisfaction [46].

The questionnaire was administered at the start of students’ clinical rotations in order to maximise participation. To ensure the best quality of data, participation was voluntary. Responses were anonymous and students were informed that only group data would be published. Students were informed about the purpose of the study by the researcher and provided with an information leaflet. Each student signed individual informed consent. The researcher discussed the items in the questionnaire with the students and was available during completion to clarify content or take queries. The authors have no academic oversight of final year students and thus the respondents were certain that there could be no negative consequences of unfavourable responses. In terms of the trustworthiness, as the authors we have no vested interests to declare in terms of its outcome.

The data was checked for completeness and inconsistencies, and captured in Microsoft Excel 2010® spreadsheets, and thereafter in IBM SPSS® Statistics 23.

Statistical methods used were as follows:

1. Categorical data was described in terms of frequency distributions and summarised in a table.
2. The data was analysed related to the specific objectives of the study, analysing items separately in relation to aspects of culture and language, for overall percentage of agreement vs. disagreement per item.
3. The final item (3b above – need for professional development) was further analysed using a suitable non-parametric test (Kruskal-Wallis test) to look for significant associations by grouping variable.

## Results

### Demographic details of the sample population

A total of 177 students were identified as eligible to complete the questionnaire. In total, 142 responses were received, which represented an 81.1% (142/175) response rate of those included, or 71.3% (142/199) of the whole 2015 final year class.

Analysis of the sample, as indicated in Table 1, showed that the group was diverse, with the majority of the students (95,1%) being South African, and 84.4% from KwaZulu-Natal (KZN). Most were KZN residents, but five other SA provinces were represented. The majority (84.5%), were SA African or SA Asian/ Indian. Several students preferred to identify themselves as African (6), Bantu (1), and Black (2), and all of these were conflated as SA African for the purposes of this analysis. Some preferred a more specific ethnicity such as isiZulu (3), or Sepedi (1). One SA student referred to herself as Chinese; just under 5% (*n* = 7) were foreign students from Botswana (one self-identified as Kalanga) and Rwanda; and two students did not complete this section.

**Table 1:** Profile of 2015 Final Year Students (*N* = 142) in Study Sample at the NRMSM

Grouping variable	N = 142	Percentage
Home Country		
South Africa	135	95.1
Botswana	6	4.2
Rwanda	1	0.7
Home Province (*n* = 135, non-SA = 7)		
KZN	114	84.4
Gauteng	4	3.0
Eastern Cape	2	1.5
Mpumalanga	6	4.4
Limpopo North West	7 2	5.2 1.5
Age		
21–24 years	97	66.2
25–29 years	40	28.2
30 years +	7	4.9
Not specified	1	0.7
Gender		
Male	55	38.7
Female	87	61.3
Ethnicity		
SA African	58	40.8
SA Asian or Indian	62	43.7
SA Coloured	3	2.1
SA White	9	6.3
(SA) Chinese	1	0.7
Botswanan	6	4.2
Rwandan	1	0.7
Do not wish to say	2	1.4
Religion		
Christianity	85	59.9
Islam	18	12.7
Hinduism	37	26.1
Traditional African Religions	0	0
Judaism	0	0
Buddhism	0	0
None	2	1.4
Do not wish to say	0	0
Home Language (alphabetically for 11 SA languages, then 2 foreign languages)		
Afrikaans	1	0.7
English	79	55.6
IsiNdebele	1	0.7
IsiXhosa	1	0.7
IsiZulu	39	27.5
Sepedi	3	2.1
Sesotho	1	0.7
Setswana	7	4.9
SiSwati	2	1.4
Tshivenda	4	2.8
Xitsonga	2	1.4
Ikalanga	1	0.7
Kinyarwanda	1	0.7
Language of Learning/Teaching (at school)		
English	139	97.9
IsiZulu	2	1.4
Sesotho	1	0.7
Number of Languages Spoken		
Monolingual (English only)	52	36.6
Bilingual	62	43.7
Multilingual (three or more)	28	19.7

(Note: Percentage figures may not add up to 100% due to rounding)

There was a female: male preponderance of 61.3 to 38.7%, and 66.2% of the respondents were in the 21–24-year age group. Several religious groups were represented. With only the official groups as per the SA Census given as options, some students designated in this analysis as Christian or Hindu preferred to be more specific with regard to religion, with a total of five specifying religion as Catholic, one as Seventh Day Adventist and one as Hare Krishna. Regarding language, the most common home language was English (55.6%), with isiZulu the second most common (27.5%), although all 11 SA official languages were represented as home languages. English was the language of learning and teaching at school for 97.9% of the sample. Of the respondents, 36.6% (*n* = 52) spoke 1 language only (English), and the remainder were bilingual (2 languages) or multilingual (3–5 languages). Additional languages other than the official SA languages spoken (sufficient for everyday interactions) were African (Shona, Kinyarwanda and Ikalanga); Asian (Tamil and Hindi); and European languages (Spanish and Portuguese).

### Students’ perceptions of concepts related to linguistic and cultural competence:

#### Role of language for HPs in SA healthcare

A total of 96.5% agreed that they were respectful of the language of the patient, and most respondents (91.5%) supported the principle of multilingualism in SA healthcare. A total of 87.4% of respondents agreed that HPs should be bilingual, whilst slightly lower percentage (83.8%) wished to improve their own language skills.

#### Cultural competence in SA healthcare

A similarly high percentage of students (96.5%) agreed that they were always respectful of their patients’ cultures. Overall, most items in this category showed a preponderance of Agree vs. Disagree responses. With regard to the use of language or cultural brokers, the percentage was considerably lower for cultural brokerage than for language interpreting (63.5 vs. 83.8%). All the respondents (100%) indicated that patients’ responses to illness, disease and death were likely to be influenced by their religious and other beliefs. The lowest percentage agreement (31.1%) in this category related to responding to the insensitive behaviours of others.

#### Students’ responsiveness

Ninety point 7% (90.7%) of students expressed a desire to ensure that principles and practices that promote cultural competence were included in the MBChB programme. In spite of a generally high percentage of agreement in the cultural competence items, 81.6% students’ perceived a need for further professional development and training to improve their knowledge and skills which they believed would help them to provide adequate services to culturally and linguistically diverse groups (Refer Table 2).

**Table 2:** Language and Cultural Competence Items; Responsiveness of Final Year Students in 2015 (*N* = 142)

Role of Language in SA Healthcare (*N* = 142)	Percentage		
	AGREE	NEUTRAL	DISAGREE
I believe that multilingualism is essential in SA healthcare	91,5	4,7	2,8
I believe that all healthcare professionals should speak at least 2 official languages	87,4	8,5	4,2
In my interactions with clients, I always respect their language	96,5	2,8	0,7
In my interactions with clients who speak languages other than my own, I attempt to improve my language skills	83,8	14,1	2,1
I use bi- or multilingual staff/ volunteers to interpret during consultations if necessary	83,8	10,6	5,6
Cultural Competence (N = 142)	Percentage		
	AGREE	NEUTRAL	DISAGREE
In my interactions with clients, I always respect their culture	96,4	3,5	0,0
I recognise that the meaning or value of health education and medical treatment may vary greatly among cultures	95,7	2,9	1,4
I accept that religion and other beliefs may influence how individuals and families respond to illness, disease and death	100,0	0,0	0,0
I seek information from individuals, families and key community informants that will help to respond to the needs and preferences of culturally and ethnically diverse groups	71,0	22,0	7,1
I use staff/ other volunteers to act as cultural brokers during consultations if necessary	63,5	24,3	12,2
I do not participate in insensitive comments or behaviours	90,2	6,3	3,5
I often respond to others’ insensitive comments or behaviours	31,1	41,3	27,5
I am aware of specific health disparities and their prevalence within local communities	78,7	17,7	3,5
I understand my clients’ cultural norms may influence communication, including greetings	97,9	0,0	2,1
I understand my clients’ cultural norms may influence communication, including eye contact	97,1	2,1	0,7
I understand the impact of culture on life activities such as gender roles	95,0	3,5	1,4
I understand the impact of culture on life activities such as customs or superstitions	78,0	17,0	4,9
I understand the impact of culture on life activities such as the use of alternative medicine	82,2	15,6	2,1
I understand the impact of culture on life activities such as the value of Western medical treatment	91,4	6,4	2,1
I provide services to those who are GLBTQ	94,3	4,3	1,4
Even though my professional or moral viewpoints may differ, I accept individuals and families as the ultimate decision makers for services and support impacting their lives	92,3	7,0	0,7
I am aware of the socio-economic and environmental risk factors that contribute to health disparities of culturally and linguistically diverse local populations	89,3	9,3	1,4
Responsiveness (N = 142)	Percentage		
	AGREE	NEUTRAL	DISAGREE
I would like the University to ensure that principles and practices promoting cultural competence are included in the medical curriculum	90,7	7,8	1,4
I need further professional development and training to improve my knowledge and skills in the provision of services and support to culturally and linguistically diverse groups	81,6	14,2	4,2

Note: Percentage figures may not add up to 100% due to rounding

The Kruskal-Wallis test (*n* = 140) for significant difference between the following independent grouping variables against the dependent variable (need for further professional development) importantly showed no significant association with gender, home language, mono- vs bi-or multilingualism, ethnicity or religion (using the groupings as per the SA Census of 2011 for the latter two variables). Although not significant, a trend emerged in the rankings for agreement for the necessity for professional development and training against monolingualism (English only) (73.36) vs. bilingualism (69.65) vs. multilingualism (67.04).

## Discussion

In response to the challenges to healthcare in the SA context, and to various SA studies which have demonstrated the barriers posed by language and culture, this study explored students’ perceptions of concepts related to linguistic and cultural competence in healthcare. This was considered important to contribute to modifying curricula in medical and HP education to respond to local needs. The contribution of language to improving cultural competence in healthcare delivery in a SA healthcare setting was considered.

The respondents comprised a diverse group, described in the results section and in Table 2. Most were South African, and from the province of KZN, an underserved and poor province in which almost 80% of the population speaks isiZulu as a mother tongue [3]. The most common home language of the respondents was English (55.6%), followed by isiZulu ((26.1%). Nearly all the students (97.9%) attended schools at which the medium of instruction was English. The commonest ethnic groups were SA Asian (43.7%), and SA African (40.8%). All the monolingual respondents (36.6%) were English-speaking, indicating that at least this percentage are unable to communicate at all with their isiZulu patients.

Steps have already been taken at this and other SA universities to address some basic African language communication skills in training, as it is widely accepted that language serves as a means to access and understand African culture [22, 47]. In this study, analysis showed generally high levels of agreement with items related to linguistic competence in the questionnaire. Respondents agreed that HPs should be bilingual (87.4%) and indicated a willingness to improve their skills in languages other than their own (83.8%).

An interesting finding, although not at a statistically significant level, was the trend shown in the analysis by number of languages spoken. This supported the fact that medical students who were bi- and multilingual were less likely than monolingual English speakers to feel the need for additional professional development to provide services to diverse groups of patients. This finding is similar to that in a Spanish language primary care context [48], where patients associated improved processes of care from physicians with higher language ability and cultural competence. In SA, in the province of KZN, a survey of cultural competence of critical care nurses in a tertiary care setting showed that nurses from non-English speaking backgrounds scored significantly higher than English-speaking nurses when interacting with largely non-English-speaking patients [49]. Such findings underpin the importance of further research in language as a facilitating factor in cultural competence in the SA healthcare environment. Whilst achieving a requisite level of language proficiency in several official languages is not possible for medical trainees due to curricular demands [50], most of the SA indigenous African languages are concentrated in specific provinces or regions. Thus mastery of more than one official language, possibly on a regional basis, should be explored as a requirement for SA HPs. For example, in KZN the dominant African language is isiZulu, which would support a requirement for HPs in this province to be proficient in isiZulu and English.

The respondents in this case study showed generally high levels of agreement with the items related to cultural competence. The highest percentage of agreement (100%) related to how religion and other beliefs may influence individuals and families’ response to illness, disease and death. Other very high levels of agreement (97.9 and 97.1%) were for an understanding of how clients’ cultural norms may influence communication, including greetings and eye contact. Both of these issues, the use of appropriate greetings for patients, and sensitivity to non-verbal cues and the patient’s body language, are stressed in basic communication teaching of process skills in the clinical method [43, 51].

In contrast with the items above, which showed high levels of agreement, the unusually low agreement rate for the item *“I often respond to others’ insensitive comments or behaviours”* may suggest that the environment for medical students to respond to racism is not enabling and be indicative of an institutional environment in which cultural competence is not valued. By inference, this finding supports the importance of creating an enabling, non-discriminatory environment in which cultural competence is emphasized and deliberately institutionalised as an organizational strategy [30]. In addition, only 63.5% of students indicated agreement with the item on the use of cultural brokers, vs 83.8% with the use of bi- or multilingual staff in language interpreting in the consultation,

With reference to the Cross Framework [17], concerns are being expressed about how some institutions in SA healthcare are perceived to show organisational behaviours in the negative range of the continuum (cultural destructiveness or incapacity) towards expressing cultural competence at an institutional level (See Fig. 1). This current study, however, explored only individual cultural competence of medical students. It is possible that the high level of agreement with the cultural competence items may be explained by acquiescence or social desirability bias. However, we would suggest that these responses indicated a level of cultural awareness and, according to the Cross framework, a level of cultural pre-competence [17] in the respondents. Cultural pre-competence implies an understanding of deficiencies and a willingness to undergo training to improve one’s abilities to deal with cross-cultural challenges [17]. This position could be explained by the fact that, in spite of the fact that cultural competence was not explicitly incorporated in training or assessment, the study population had been trained using a patient-centred model [43] and sensitised to the incorporation of the patient’s perspective. It is therefore likely that their responses may have been influenced by this and other experiential exposures in the curriculum. Using a person-centred approach [45] implies that each patient is valued as an individual, and that medical students have appropriate knowledge, skills and behaviours to interact with each patient appropriately. This links to the concept of cultural humility [12] (a lifelong commitment to reflection and self-critique in the use of practices such as patient-focused interviewing and care), which is believed to be an attainable goal in multicultural education.

The pre-competent position would be supported by the majority of respondents expressing high levels of agreement with the two items deemed to indicate respondents’ responsiveness: expressing high levels of agreement with the inclusion by the University of principles and practices promoting cultural competence in the medical curriculum, and with the need to improve knowledge and skills to provide services to culturally and linguistically diverse groups.

This latter item (need to improve knowledge and skills) was analysed further for association by grouping variable to investigate whether any particular grouping expressed a greater level of agreement, and an important negative finding was that no significant associations were found; thus students’ perceived need for further professional development and training was equally expressed across the sample and not influenced by ethnicity, religion or home language. The finding further supports the fact that each HP, in fact, reflects aspects of a *“culture”* which relates to his or her context and life experience, and emphasises the need to refrain from stereotyping by grouping variable in HP education and/or any other setting. This is considered important in view of SA’s past history of racial discrimination, the politicisation of language and the fact that the legacy of racial segregation is that *“most cultural/ethnic groups in SA still have limited interaction with other groups on a social level”* ([49], p.4).

### Implications for education and future research

To respond to the perceived need for additional professional development and to improve the responsiveness of the curriculum to local needs, it is recommended that specific learning objectives related to cultural competence should be included in medical training. Increased emphasis on intercultural communication, multiculturalism and communication ethics in basic communication courses should be encouraged [24] to maximise effectiveness of HPs in resource-challenged healthcare environments. The lower agreement expressed with the item on the use of cultural brokers vs that regarding the use of bi- or multilingual staff in language interpreting in the consultation, suggests that the use of cultural brokers in training [11] should be explored as a means to sensitise students to the inclusion of cultural factors in the medical interview. The diverse nature of the respondents should be seen as a resource for learning. Providing opportunities for students to explore their own cultural awareness, for example by the use of self-reflection checklists, may increase awareness of the influence of their own and other cultures and cultural biases [52] on interactions in the healthcare context. Whilst much emphasis in SA is placed on African culture, this would also allow deeper exploration of additional cultural issues, for example those related to gender roles, religion and sexual orientation in health. Understanding of concepts related to both individual and institutional cultural competence would improve students’ insights into their relevance in responding to the challenges, particular those related to culture, in SA healthcare.

Research and development in cultural competence for medical and other HP education in SA contexts from individual to institutional and system levels is recommended. This should include research into how cultural competence is facilitated by language in the SA context. In order to respond better to the needs of local communities, research at a community level would assist in gaining an understanding of patients’ perspectives and satisfaction with the cultural competence of HPs and healthcare organisations serving the SA public.

### Limitations of the study

Only individual and not institutional cultural competence was explored in the study. The items related to cultural competence were based on items in assessment tools from a non-Southern African context but were adapted for use in this exploratory study. The findings relate to self-assessment of students’ perceptions at one medical school only, and are not generalizable, but it is hoped that the study findings will be generative, promote discussion and suggest opportunities for further research on the teaching and learning of cultural competence for medical and other HP students in similar SA healthcare contexts.

## Conclusion

This study explored perceptions of concepts relating to linguistic and cultural competence in final year SA medical students at one institution. It raised the importance of language and culture in the SA healthcare environment. Students’ perceptions on language use and cultural competence indicated high levels of agreement in general, but nonetheless, students indicated a need for professional development in cultural competence and provision of services to culturally and linguistically diverse groups. Integrating culture and language teaching and learning may be a facilitating factor in developing cultural competence for medical students and improving healthcare and outcomes in the SA healthcare context.

## Abbreviations

CDC: Centres for Disease Control
HP: Health professions/ health professional
KZN: KwaZulu-Natal
MBChB: Bachelor of Medicine, Bachelor of Surgery
NRMSM: Nelson R Mandela School of Medicine
SA: South Africa/ South African
UKZN: University of KwaZulu-Natal
